
JOURNAL INFORMATION
==============================
NLM Title Abbreviation: Tex Med J (Austin)
NLM Journal ID: 21130590R

Texas Medical Journal

ISSN: 0892-8495
Publisher: Biomedical Journal Digitization Project

ARTICLE INFORMATION
==============================
PMCID: PMC9591540
Article version: 1
Subjects: Books and Magazines

Essentials of Physical Diagnosis of the Thorax

Print publication date: 1900 Oct
Volume: 16
Issue: 4
First page: 181
Last page: 181
License: This work is free of known copyright restrictions. For more information, please see PMC Back Issue Digitization.
License URL: https://creativecommons.org/publicdomain/mark/1.0/

==============================

enough to meet the demands of schools, and the arrangement of the subject matter is simple and convenient. There is no work on the market more valuable to the student of medicine than this one.

T. J. B.

---

Essentials of Physical Diagnosis of the Thorax.—By Arthur M. Corwin, A. M., M. D., Instructor of Physical Diagnosis in Rush Medical College, etc. Third edition; revised and enlarged. 220 pages. Price, $1.25. Philadelphia: W. B. Saunders, 925 Walnut Street. 1899.

This little book was intended in its first edition to supply the demands of the class at Rush, but later the author yielded to a wider demand and enlarged the volume into its present form. It is a succinct presentation of the subject, and is especially adapted to students and practitioners who desire to review the whole subject in a very short time. T. J. B.

---

Diabetis Mellitus—Its Detection and Successful Treatment.—The Journal has received a broachure under the above title, issued by the Charles Roome Parmele Co., 36 Platt Street, New York, the manufacturers and proprietors of Arsenauro and Murcano. (See advertisement on inside page.) It is a neatly printed pamphlet, containing very able and well written articles on the subject and illustrated with several colored plates showing the test for sugar in urine by the Fehling solution, and other cuts, Stern's Urino-Glucosometer and Einhorn's Saccharometer, Crystals of Phenyl Glucosazone as shown by Phenylhydrazin Test, etc. In the treatment of diabetis in a large number of cases reported in the papers referred to, and which are reprints from the *New York Medical Journal* (papers by Heinrich Stein, Ph. D., M. D., and Geo. D. Barney, M. D.), and from the *International Medical Magazine* (paper by Alexander W. Beck, M. D.), and several others, the two preparations above named were used, either exclusively or in connection with other remedies, and the results were remarkable. The Charles Roome Parmele Co. will mail a copy of this valuable pamphlet free to any one on receipt of a postal card, mentioning the "Red Back."

---

### Publisher's Department.

"Vin Mariani" is essentially the brain and nerve tonic of those who have talent and genius. These it is who compose the great army of intellectual workers, and the ravages made upon their nervous systems by the demands made upon them are at times truly appalling. This damage and consequent drain
